# Influence of Sulfur Fumigation on the Chemical Constituents and Antioxidant Activity of Buds of *Lonicera japonica*

**DOI:** 10.3390/molecules191016640

**Published:** 2014-10-15

**Authors:** Ai-Li Guo, Liang-Mian Chen, Yan-Min Wang, Xiao-Qian Liu, Qi-Wei Zhang, Hui-Min Gao, Zhi-Min Wang, Wei Xiao, Zhen-Zhong Wang

**Affiliations:** 1 Institute of Chinese Materia Medica, China Academy of Chinese Medical Sciences, Beijing 100700, China; E-Mails: guoaililu@126.com (A.-L.G.), chris-clm@163.com (L.-M.C.), wangyanmin@126.com (Y.-M.W.), lianyu1127@126.com (X.-Q.L.), zhangqw1955@163.com (Q.-W.Z.); 2 National Engineering Laboratory for Quality Control Technology of Chinese Herbal Medicine, Beijing 100700, China; 3 Jiangsu Kanion Pharmaceutical Co. Ltd., Lianyungang 222001, China; E-Mails: wzhzh-nj@tom.com (W.X.); wzhzh-nj@tom.com (Z.-Z.W.)

**Keywords:** *Lonicera japonica* Thunb., sulfur fumigation, sulfur dioxide residual, secologanic acid, antioxidant activity

## Abstract

*Lonicera japonica flos* is widely used as a pharmaceutical resource and a commonly-employed ingredient in healthy food, soft beverages and cosmetics in China. Sometimes, sulfur fumigation is used during post-harvest handling. In this study, a comprehensive comparison of the chemical profile between sun-dried and sulfur-fumigated samples was conducted by HPLC fingerprints and simultaneous quantification of nine constituents, including secologanic acid, along with another eight usually-analyzed markers. Secologanic acid was destroyed, and its sulfonates were generated, whereas caffeoylquinic acids were protected from being oxidized. The residual sulfur dioxide in sulfur-fumigated samples was significantly higher than that in sun-dried samples, which might increase the potential incidence of toxicity to humans. Meanwhile, compared with sun-dried samples, sulfur-fumigated samples have significantly stronger antioxidant activity, which could be attributed to the joint effect of protected phenolic acids and flavonoids, as well as newly-generated iridoid sulfonates.

## 1. Introduction

Sulfur fumigation as an increasingly commonly-employed post-harvest process technique for some traditional Chinese Medicines (TCMs) has attracted more and more attention in the last decade due to its potential detrimental effect on the safety and efficacy of sulfur-fumigated TCMs [1,2]. Although the China Food and Drug Administration (CFDA) has promulgated that the residue limit of sulfur dioxide should not be more than 400 mg/kg for 12 TCMs, including *Achyranthis bidentatae radix*, *Dioscoreae rhizoma*, *Puerariae thomsonii radix*, and so on, and less than 150 mg/kg for all other TCMs [3], some herbal farmers and producers still continue to use sulfur fumigation for pesticide purposes. Besides residual sulfur dioxide, understanding the chemical changes of the key active ingredients in the herbs induced by sulfur fumigation is also crucially important. Such chemical alteration of TCMs has been evidenced undoubtedly by increasing numbers of studies, involving sulfur-fumigated white peony roots [4,5,6], white ginseng [7], *Angelicae dahuricae radix* [8], *Fritillariae thunbergii bulbus* [9], *Chrysanthemi flos* [10,11] and *Codonopsis radix* [12].

*Lonicera japonica flos* (LJF), well-known as Jin Yin Hua in China, the dried buds of *Lonicera japonica* Thunb., is widely used as a pharmaceutical resource for the treatment of various viral diseases, such as SARS, H7N9 virus and infections [13,14]. Meanwhile, it is also used as a frequently-used ingredient in healthy food, soft beverages and cosmetics for its specific benefits, such as antibacterial, anti-inflammatory, antiviral, anti-endotoxin, antipyretic and antioxidative activities [15]. Essential oils, organic acids, flavones and iridoids have been reported as the active constituents with some potential pharmacological effects. The influence of sulfur fumigation on the volatile components of LJF was reported by GC × GC–TOF/MS analysis [16]. However, no systematic investigations are available regarding the effect of sulfur fumigation on the non-volatile components of LJF, except our previous study in which three new secoiridoid sulfonates were isolated and identified from sulfur-fumigated LJF [17]. To comprehensively understand the influence of the sulfur fumigation process on non-volatile components and the potent bioactivity of LJF, a comparison of the relative and absolute amounts of the main chemical constituents (chlorogenic acid and its derivatives, flavones and iridoids), residual sulfur dioxide and the antioxidant activity between sun-dried and sulfur-fumigated LJF samples was carried out. The present study can provide some evidence for the effective and safe application of LJF and its related functional foods.

## 2. Results and Discussion

### 2.1. Qualitative HPLC and LC-ESI/MS^n^ Analyses

#### 2.1.1. The Chemical Difference between Sun-Dried and Sulfur-Fumigated LJF Samples

**Figure 1:**
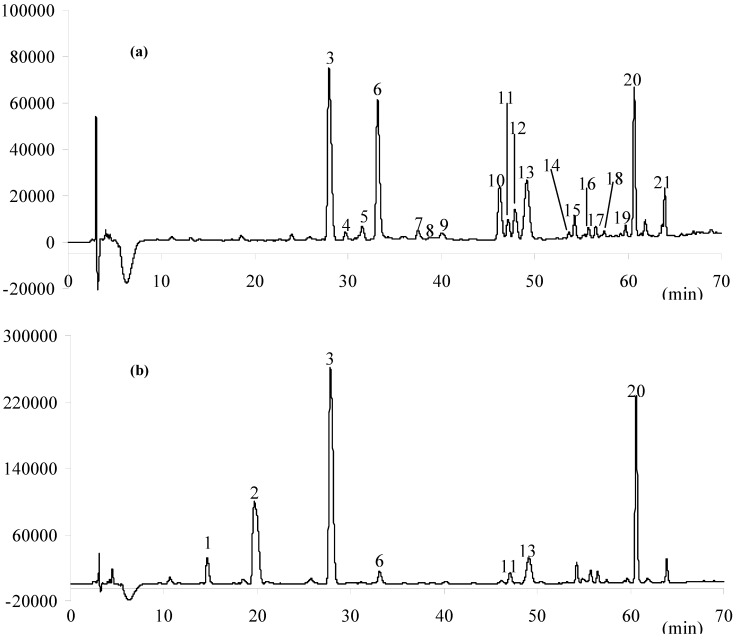
HPLC chromatograms of *Lonicera japonica flos* (LJF) samples collected in Shandong province for the qualitative analysis. (**a**) Sun-dried; (**b**) Sulfur-fumigated. The peak number refers to the compound number shown in Table 1.

Under the optimized HPLC conditions, 16 batches of sun-dried or sulfur-fumigated LJF samples, as well as the reference compounds were examined. The typical chromatograms of sun-dried and sulfur-fumigated LJF samples are shown in Figure 1. It was found that secologanic acid (Peak **6**) was decreased sharply in sulfur-fumigated samples, compared with sun-dried samples. Meanwhile, there are some chromatographic peaks (Peaks **1** and **2**) with shorter retention times than that of chlorogenic acid (Peak **3**), detected in the sulfur-fumigated samples, but not in the sun-dried samples (Figure 1, Table 1). The chemical alternation that was proposed to be involved in the reactions of esterification or ring-opening, followed by the addition reaction of naturally occurring secologanic acid, was in good agreement with the previous investigation on sulfur-fumigated LJF samples collected in May 2007 [17]. It showed that the chemical transformation induced by sulfur fumigation in LJF samples was reproducible and representative.

**Table 1:** The identified compounds in sulfur-fumigated and sun-dried LJF samples by HPLC and LC-MS^n^.

No.	t_R_ (min)	UV λ_max_ (nm)	M w	Negative	Positive	MS^2^	MS^3^	Identified Compounds	SF ^a^	SD ^b^
**1**	16.3	247	438	437 [M − H]^−^		367 [M−SO_3_H−H]^−^		1	+	−
**2**	22.3	241	470	469 [M − H]^−^		451 [M−H_2_O−H]^−^	387 [451−SO2]^−^	2	+	−
**3**	30.0	244/325	354	353 [M − H]^−^	377 [M + Na]^+^	192 [M−caffeoyl−H]^−^		chlorogenic acid	+	+
**4**	32.2	247	376	375 [M − H]^−^	399 [M + Na]^+^	213 [M−glc−H]^−^		loganic acid	−	+
**5**	34.1	245	390	389 [M − H]^−^	413 [M + Na]^+^			loganin isomer	+	+
**6**	35.8	246	374	373 [M − H]^−^	397 [M + Na]^+^	194 [M−glc−OH−H]^−^		secologanic acid	+	+
**7**	39.1	250/311	698	697 [M − H]^−^	721 [M + Na]^+^			not identified	+	+
**8**	40.6	250	390					loganin	+	+
**9**	41.6	251	358					sweroside	+	+
**10**	49.9	249	388	433 [M + HCOO]^−^	427 [M + K]^+^			epivogeloside	−	+
**11**	50.5	247	404	403 [M − H]^−^	427 [M + Na]^+^	371 [M−CH_3_OH]^−^ 265 [M−glc + Na]^+^	233 [M−glc−CH_3_OH + Na]^+^	secoxyloganin	+	+
**12**	51.5	254	388	433 [M + HCOO]^−^	427 [M + K]^+^			vogeloside	−	+
**13**	52.6	245	388	433 [M + HCOO]^−^	411 [M + Na]^+^ 427 [M + K]^+^	225 [M−glc−H]^−^		secologanin	+	+
**14**	55.7	254/337	730	729 [M − H]^−^	753 [M + Na]^+^			not identified	+	+
**15**	56.1	254/353	610	609 [M − H]^−^	633 [M + Na]^+^	301 [M−glc−rham−H]^−^	255	rutin	+	+
**16**	57.3	254/327	464	463 [M − H]^−^	465 [M + H]^+^	301 [M−glc−H]^−^		hyperoside	+	+
**17**	58.0	252/344	448	447 [M − H]^−^	449 [M + H]^+^	285 [M−glc−H]^−^		luteoloside	+	+
**18**	59.1	252/347	594	593 [M − H]^−^		285 [M−glc−rham−H]^−^		lonicerin	+	+
**19**	60.7	251/323	516	515 [M − H]^−^		353 [M−caffeoyl−H]^−^		isochlorogenic acid B	+	+
**20**	61.7	246/327	516	515 [M − H]^−^	539 [M + Na]^+^	353 [M−caffeoyl−H]^−^ 377 [M−caffeoyl + Na]^+^	175 [M−caffeoyl−H−178]^−^ 215 [M−2caffeoyl + Na]^+^	isochlorogenic acid A	+	+
**21**	64.7	249/327	516	515 [M − H]^−^	539 [M + Na]^+^	353 [M−caffeoyl−H]^−^		isochlorogenic acid C	+	+

^a^ Sulfur-fumigated; ^b^ Sun-dried.

**Figure 2:**
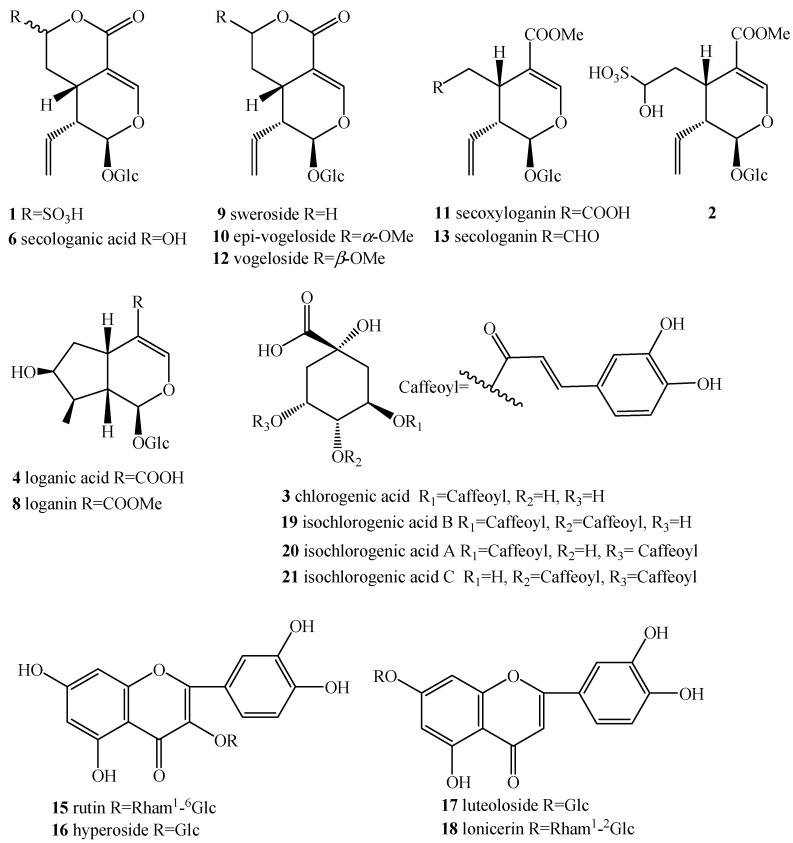
Chemical structures of the main compounds in LJF samples.

Previous studies dealing with the screening and characterization of the active components in LJF by LC-MS^n^ have been reported [18,19,20,21]. Therefore, a detailed discussion of the fragmentation pattern of each compound class of caffeoylquinic acids, flavonoids and iridoids is beyond the scope of this paper. LC-ESI/MS^n^ analysis in this study is aimed to further distinguish and confirm the varying or un-varying chromatographic peaks in sulfur-fumigated or sun-dried LJF samples. Iridoid glycosides can be better detected not only in positive ESI mode, but also in negative mode, while caffeoylquinic acids and flavonoids gave more information only in negative mode. The target peak identification was conducted by comparing the retention time and on-line ultraviolet spectra with those of reference substances, as well as mass spectra and fragmentation rules (Table 1). As a result, 21 main constituents were identified (Figure 2), including chlorogenic acid (Peak **3**) and three dicaffeoylquinic acids (Peaks **19**–**21**), ten iridoid glycosides (Peaks **1**, **2**, **4**, **6** and **8**–**13**) and five flavonoids (Peaks **14**–**18**). In particular, secologanic acid sulfonates (**1** and **2**) generated by the sulfur fumigation process gave the characteristic fragmentation pattern, representing the loss of neutral SO_2_ deduced by a prominent loss of 64 Da from the deprotonated molecular ion of [M − H]^−^, which could be used to differentiate whether the LJF sample was sulfur-fumigated or not.

#### 2.1.2. Multivariate Statistical Analysis

Peaks that existed in the samples with reasonable height and good resolution were assigned as characteristic peaks for the identification of the LJF samples. The area of each peak was recorded (the peak number refers to Figure 1), and the data were analyzed by multivariate statistical analysis (SIMCA-P+11) in order to visually demonstrate the resemblance and difference of the sulfur-fumigated and sun-dried samples, as well as the easily-influenced constituents during the sulfur fumigation process. The results of principal component analysis (PCA) of the HPLC data obtained from the samples (Nos. 3–8 and 11–16) collected from the same growing area (excluding the effect of the different habitats on the quality of LJF samples) showed that all samples were clearly classified into the sulfur-fumigated and sun-dried types. The sulfur-fumigated samples (Nos. 3–8) were distributed at the right of the plot, while the sun-dried samples (Nos. 11–16) were at the left of the plot (Figure S1a). The results of partial least squares (PLS) displayed a similar plot. The sulfur-fumigated samples were distributed at the right of the plot, whereas the sun-dried samples were at the left (Figure S1b). Variable importance in the projection (VIP) indicated that chlorogenic acid (Peak **3**), secologanin (Peak **13**), isochlorogenic acid A (Peak **20**), secologanic acid (Peak **6**) and its sulfonates (Peak **2**) as well as Peak **5** were easily varied during postharvest processing (Figure S1c, VIP values >1).

### 2.2. Quantification of Main Phenolic Acids, Flavonoids and Iridoids in the LJF Samples

A number of diverse compounds, such as phenolic acids, flavonoids and iridoid glycosides, have been isolated from LJF samples [15]. Generally, chlorogenic acid and flavonoids (lutin and luteoloside) were chosen as marker compounds for the quality evaluation and standardization of LJF and its preparations [13]. As another type of pharmacologically important constituent, major iridoid glycosides have not been paid enough attention for the quality evaluation of LJF. A few investigations were reported involving in the quantitative analyses of iridoid glycosides, including sweroside, 7-epivogeloside, secoxyloganin, dimethyl-secologanoside, centauroside and loganin, along with phenolic acids and flavonoids, by HPLC-DAD-ELSD [22] and HPLC-DAD-MS [18,19,23]. However, from our study mentioned above, secologanic acid was the most abundant iridoid, and its content was far higher than those of sweroside and loganin, which were commonly used as the representatives of iridoid glycosides in LJF samples. Furthermore, secologanic acid is easily destructed and generated its sulfonates in the sulfur fumigation process. Due to the poor stability of the derived sulfonates, it is not possible to obtain enough amounts of this class of compounds for quantitative analysis of LJF samples. Therefore, secologanic acid is one of the most suitable marker compounds for the differentiation of sulfur-fumigated and non-fumigated LJF samples. In the following investigation, simultaneous determination of nine constituents by HPLC-DAD, including chlorogenic acid, secologanic acid, sweroside, loganin, rutin, luteoloside, isochlorogenic acid A, B and C, was described.

**Figure 3:**
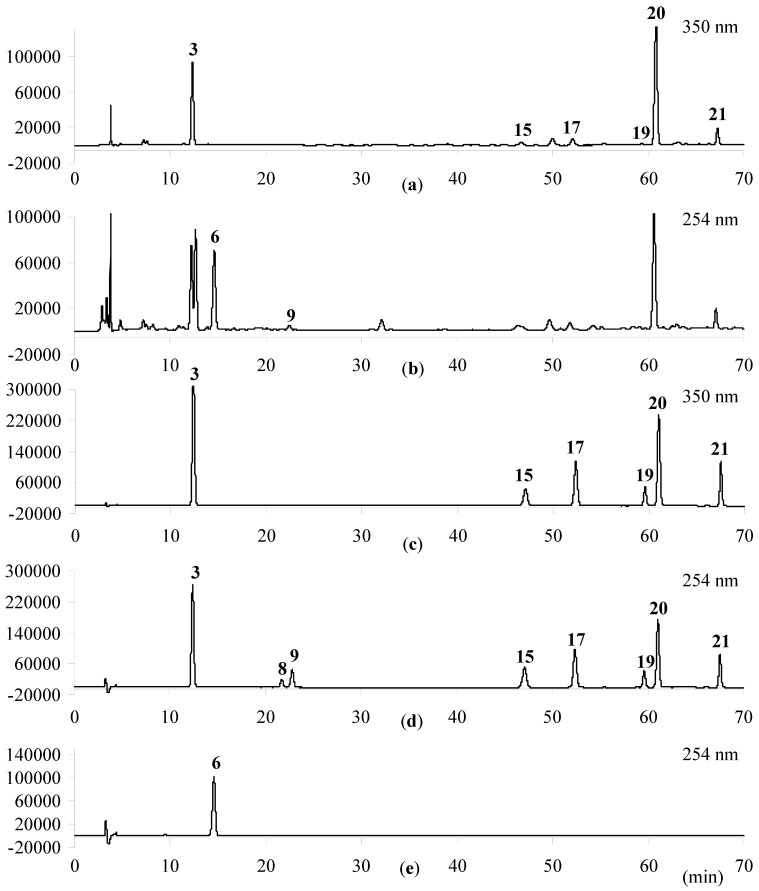
HPLC chromatograms of LJF samples collected in Jiangsu province (**a**,**b**) and mixed reference substances (**c**–**e**). (a) samples detected at 350 nm; (b) samples detected at 254 nm; (c) mixed reference substances of chlorogenic acid (**3**), rutin (**15**), luteoloside (**17**), isochlorogenic A (**20**), isochlorogenic B (**19**) and isochlorogenic C (**21**), detected at 350 nm; (d**)** mixed reference substances of loganin (**8**) and sweroside (**9**), detected at 254 nm. (e) Reference secologanic acid (**6**), detected at 254 nm.

For more satisfactory chromatographic separation of the analytes, the gradient elution program was optimized, as described in Section 3.4. 254 nm was employed to detect iridoid glycosides (secologanic acid, loganin and sweroside), and 350 nm was chosen to simultaneously analyze phenolic acids (chlorogenic acid, isochlorogenic acid A, isochlorogenic acid B and isochlorogenic acid C) and flavonoids (rutin and luteoloside), respectively. The typical HPLC chromatogram is shown in Figure 3. It is observed that nine compounds could be completely separated from the neighboring chromatographic peaks in a single run.

Using the established method, 16 batches of LJF samples were analyzed, and the contents of the determined compounds are shown in Table 2. Chlorogenic acid (**3**), secologanic acid (**6**) and isochlorogenic acid A (**20**) were abundant in all samples with a relatively high content of 0.299%–4.54%, 0.196%–2.63% and 0.469%–2.58%, while sweroside (**9**), rutin (**15**), luteoloside (**17**), isochlorogenic acid B (**19**) and C (**21**) were presented relatively low, less than 0.082%, 0.098%, 0.085%, 0.040% and 0.26%, respectively. Loganin was not determined in LJF samples due to being below the LOQ. Compared with sun-dried samples, secologanic acid has a far lower amount in sulfur-fumigated samples, whereas caffeoylquinic acids (**3**, **19**–**21**) were higher in sulfur-fumigated samples than in sun-dried samples. For the contents of flavonoids (rutin and luteoloside), no significant influence was observed with sulfur fumigating for LJF. As the literature displayed, the main o-diphenolic compounds in LJF were chlorogenic acid and isochlorogenic acids [24]. The polyphenol oxidase (PPO) enriched in the damaged tissues of fresh LJF, which oxidize caffeoylquinic acids to pigments in processing, is involved in the enzymatic browning leading to severe loss of this type of compound [25]. In our opinion, the sulfur fumigation process by burning sulfur can effectively inhibit the enzymatic activation of PPO and protect dicaffeoylquinic acids from being oxidized, which also suggested that it is necessary for the selection of an alternative post-harvest process technology of LJF to inhibit enzymatic browning and reduce the loss of phenolic compounds.

### 2.3. Residual Sulfur Dioxide

The residual sulfur dioxide (SO_2_) in the sun-dried and sulfur-fumigated LJF samples was determined by distillation in acidic aqueous solution and iodine titration [13]. The results suggested that residual SO_2_ in the sulfur-fumigated LJF is much higher than that in the sun-dried sample (Table 2), which is also far higher than the residue limit SO_2_ promulgated by CFDA, not to exceed 150 mg·kg^−1^ in sulfur-fumigated samples. Actually, almost all TCMs roughly processed by sulfur fumigation present a high level of residual SO_2_, which might increase the potential incidence of toxicity.

### 2.4. The Antioxidant Capacity

**Figure 4:**
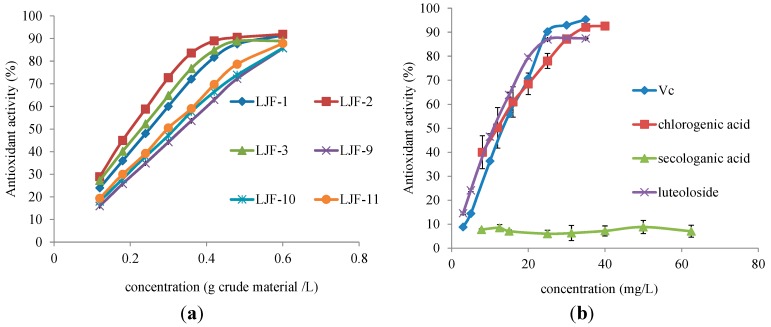
The antioxidant capability of sulfur-fumigated samples, sun-dried samples and reference compounds (n = 3). (**a**) The percentage of free radical scavenging of LJF samples at concentrations of 0.12, 0.18, 0.24, 0.30, 0.36, 0.42, 0.48 and 0.60 g crude material/L. (**b**) The percentage of free radical scavenging of reference compounds. The concentration was as follows: 3, 5, 10, 15, 20, 25, 30, 35 mg/L for Vc and luteoloside (**17**); 8, 12, 16, 20, 25, 30, 35, 40 mg/L for chlorogenic acid (**3**); and 7.8, 12, 15, 25, 31, 40, 50, 62 mg/L for secologanic acid (**6**).

The antioxidant activity of LJF plays an important role in its therapeutic effect for various diseases. Therefore, it was selected as the bioactive index for comprehensively evaluating the difference of the sulfur-fumigated and sun-dried LJF samples. Using the most employed DPPH (2,2-diphenyl-1-picrylhydrazyl) method, the antioxidant effect of the aqueous ethanol (1:1, v/v) extract of sulfur-fumigated (Nos. 1–3) and sun-dried samples (Nos. 9–11), as well as pure compounds chlorogenic acid (**3**), secologanic acid (**6**), luteoloside (**17**) and ascorbic acid (Vc) was estimated. As shown in Figure 4 and Table 3, all of the extracts showed a good radical scavenging activity. The sulfur-fumigated samples had lower EC_50_ values than those of the corresponding sun-dried samples, and a significant difference was found (*p* < 0.05). Phenolic acids and flavonoids were the major natural antioxidants in LJF extract, and iridoid glycosides, such as sweroside (**9**) and centauroside, were inactive in the presence of DPPH free radicals [20]. The antioxidative activity of the determined reference Compounds **3**, **6** and **17** agreed with this trend described above. Chlorogenic acid (**3**) and luteoloside (**17**) possessed higher or similar activity, corresponding to the classical antioxidant, ascorbic acid, whereas secologanic acid (**6**) had no activity with respect to DPPH free radicals.

**Table 2:** The contents of marker compounds and SO_2_ residual in the sulfur-fumigated and sun-dried LJF samples.

Samples	Growing Area	Collecting Time	Drying Method	Marker Compounds (%)								SO_2_ Residual (mg/g)
				3	6	9	15	17	19	20	21	
**1**	Shandong	05/2012	Sulfur-fumigated	4.00	0.196	0.0212	0.0918	0.0646	0.0196	1.50	0.128	1.954 ± 0.103
**2**	Shandong	05/2012	Sulfur-fumigated	4.35	0.228	0.0306	0.0838	0.0395	0.0233	1.49	0.152	1.611 ± 0.054
**3**	Jiangsu	05/2013	Sulfur-fumigated	3.17	0.765	0.0661	0.0509	0.0609	0.0222	2.07	0.177	
**4**	Jiangsu	05/2013	Sulfur-fumigated	3.50	0.825	0.0650	0.0528	0.0704	0.0265	2.11	0.188	
**5**	Jiangsu	05/2013	Sulfur-fumigated	4.54	0.607	0.0490	0.159	0.0841	0.0397	2.58	0.252	
**6**	Jiangsu	05/2013	Sulfur-fumigated	3.67	0.744	0.0570	0.0454	0.0658	0.0309	2.45	0.188	
**7**	Jiangsu	05/2013	Sulfur-fumigated	3.95	0.764	0.0634	0.0362	0.0628	0.0319	2.38	0.175	
**8**	Jiangsu	05/2013	Sulfur-fumigated	1.39	1.25	0.0506	0.0363	0.0305	0.0210	1.87	0.108	
**9**	Shandong	05/2012	Sun-dried	2.47	2.63	0.0816	0.0594	0.0459	0.0227	1.09	0.147	0
**10**	Shandong	05/2012	Sun-dried	2.55	2.12	0.0715	0.0979	0.0847	0.0281	0.904	0.149	0
**11**	Jiangsu	05/2013	Sun-dried	1.33	1.23	0.0466	0.0417	0.0675	0.0211	1.42	0.181	
**12**	Jiangsu	05/2013	Sun-dried	2.39	1.64	0.0544	0.0486	0.0827	0.0257	1.63	0.215	
**13**	Jiangsu	05/2013	Sun-dried	0.299	0.793	0.0415	0.0299	0.0447	0.0103	0.469	0.0708	
**14**	Jiangsu	05/2013	Sun-dried	2.09	1.47	0.0423	0.0310	0.0652	0.0289	1.91	0.209	
**15**	Jiangsu	05/2013	Sun-dried	0.389	0.853	0.0430	0.0210	0.0450	0.0125	0.655	0.0787	
**16**	Jiangsu	05/2013	Sun-dried	0.867	1.63	0.0602	0.0369	0.0541	0.0180	1.20	0.120	

**Table 3:** The EC_50_ values of sulfur-fumigated LJF, sun-dried LJF and reference compounds.

LJF Sample No./ Reference Compounds	EC_50_^a^
**1**	0.23 ± 0.0017
**2**	0.19 ± 0.0032
**3**	0.21 ± 0.0074
**9**	0.31 ± 0.0045
**10**	0.29 ± 0.011
**11**	0.28 ± 0.014
**ascorbic acid (Vc)**	0.064 ± 0.007
**chlorogenic acid (3)**	0.032 ± 0.005
**secologanic acid (6)**	0.19 ± 0.08
**luteoloside (17)**	0.22 ± 0.0003

^a^ g of crude material/L for LJF samples and mmol/L for the reference compounds.

In order to determine further the possible relationship of EC_50_ values and the contents of the main compounds in LJF, bivariate correlation analysis was carried out by the SPSS 17.0 software. The results indicated that chlorogenic acid (**3**) and isochlorogenic acid A (**20**) are highly correlated to the antioxidant capacity of LJF. The Pearson correlation coefficients were as follows: chlorogenic acid (−0.798, significant 0.057, two-tailed) and isochlorogenic acid A (−0.737, significant 0.095, two-tailed). The more phenolic acids in the LJF sample, the lower the EC_50_ value. Such results also provided some evidence for the stronger antioxidant activity of sulfur-fumigated LJF samples with the relatively higher contents of dicaffeoylquinic acids. In a word, although an obvious chemical difference was observed between sulfur-fumigated and sun-dried samples, their antioxidant activity was not decreased. Instead, to some extent, it would be increased. This might result from the protected phenolic acids and flavonoids, as well as newly-generated iridoid sulfonates with more antioxidant activity during the sulfur fumigation process.

## 3. Experimental Section

### 3.1. Chemicals, Reagents and Herb Materials

Four batches of LJF were collected in May 2012, from Jinan, Shandong province, and another twelve batches were collected in May 2013, from Lianyungang, Jiangsu province, in China. All of the samples were identified by professor Zhi-Min Wang (Institute of Chinese Materia Medica, China Academy of Chinese Medical Sciences, Beijing, China) as the buds of *L. japonica* Thunb. The fresh buds were processed as follows: the sun-dried samples were obtained by drying in the Sun, and the sulfur-fumigated samples were obtained by fumigating with SO_2_ gas from burning sulfur and then drying in the shade.

The quantitative reference compounds of chlorogenic acid (**3**, Lot: 110753-200413), loganin (**8**, Lot: 111640-200401), sweroside (**9**, Lot: 111742-200501) and rutin (**15**, Lot: 100080-200707), were purchased from the National Institute for Food and Drug Control (Beijing, China). Luteoloside (**17**, Lot: 20130521), isochlorogenic acid B (**19**, Lot: 20130121), isochlorogenic acid A (**20**, Lot: 20130121) and isochlorogenic acid C (**21**, Lot: 20130121) were purchased from Shanghai Yuanye Bio-Technology Co., Ltd. (Shanghai, China). Secologanic acid (**6**) was isolated from the sun-dried LJF sample, and its structure was identified based on the spectral data. The chemical structures of these compounds mentioned above are shown in Figure 2. Ascorbic acid (Vc) was purchased from Sinopharm chemical reagent Co., Ltd. (Beijing, China) (Lot: 20110922). 2,2-diphenyl-1-picrylhydrazyl (DPPH free radical, Lot: D19Y029) was purchased from Alfa Aesar Co., Ltd (Tianjin, China). The reference iodine solution with a concentration of 0.0496 mol/L (I_2_ (1/2), Lot: 12012) was purchased from National Institute of Metrology (Beijing, China).

HPLC-grade methanol (Fisher, Waltham, MA, USA) and acetonitrile (Fisher, Waltham, MA, USA), as well as ultra-purity H_2_O were used for HPLC and LC-MS analyses. All other reagents were of analytical grade and obtained from Beijing Chemical Company (Beijing, China).

### 3.2. Sample Preparation

#### 3.2.1. LJF Sample Solution for HPLC and LC-MS Analysis

Each of the powdered LJF samples (60 mesh, 0.4 g for the qualitative analysis, 0.2 g for the quantitative analysis) was extracted by ultrasonication for 20 min at room temperature with aqueous methanol (MeOH/H_2_O 1:1), 50 mL, for qualitative HPLC and LC-MS analyses or aqueous ethanol (EtOH/H_2_O 1:1), 20 mL, for quantitative HPLC analysis. The extract was centrifuged at 6985× *g* for 10 min, and 10 µL of the supernatant were used for qualitative and quantitative analyses.

#### 3.2.2. Reference Solution

For the quantitative determination of nine constituents in LJF samples, an accurately weighed amount of each reference substance was mixed and dissolved in 10 mL of methanol to obtain a stock solution with a concentration of 336 mg/L for chlorogenic acid (**3**), 480 mg/L for secologanic acid (**6**), 40 mg/L for loganin (**8**), 50 mg/L for sweroside (**9**), 48 mg/L for rutin (**15**), 56 mg/L for luteoloside (**17**), 40 mg/L for isochlorogenic acid B (**19**), 200 mg/L for isochlorogenic acid A (**20**) and 70 mg/L for isochlorogenic acid C (**21**), respectively.

#### 3.2.3. LJF Samples and Reference Solutions for the Antioxidant Capacity Assay

For the assay on the antioxidant capacity, the powdered LJF samples were extracted by ultrasonication for 20 min at room temperature with aqueous ethanol (EtOH/H_2_O, 1:1) to obtain a concentration of 0.60 g crude material/L. The extract was filtered and diluted with EtOH/H_2_O (1:1) to eight different concentrations (0.12–0.60 g crude material/L).

For the antioxidant capacity of the reference compounds, an accurately weighed amount of each reference substance was mixed and dissolved in 25 mL of methanol to obtain a stock solution with a concentration of 35 mg/L for Vc, 40 mg/L for chlorogenic acid (**3**), 35 mg/L for luteoloside (**17**) and 62 mg/L for secologanic acid (**6**), respectively. The stock solution was diluted with MeOH to eight concentrations (35–3.0 mg/L for Vc and luteoloside, 40–8.0 mg/L for chlorogenic acid, 62–7.8 mg/L for secologanic acid).

### 3.3. Qualitative HPLC and LC-ESI/MS Analysis

To compare the chemical difference between the sun-dried and sulfur-fumigated LJF samples, HPLC analysis was performed with a Shimadzu HPLC system (Shimadzu, Kyoto, Japan) equipped with an LC-20 quaternary pump, a diode array detector, an autosampler and a column compartment. The samples were analyzed on a Kromasil-C_18_ column (4.6 mm × 250 mm, 5 μm). The mobile phase consisted of water (adjusted pH to 2.0 with HCOOH, A) and acetonitrile (B), and the linear gradient was as follows: 0–20 min (3%–8% B), 20–45 min (8%–15% B), 45–60 min (15%–24% B), 60–70 min (24%–40% B). The flow rate was at 1.0 mL/min. The DAD detector recorded UV spectra in the range from 190 nm to 400 nm, and the HPLC chromatogram was monitored at 254 nm.

To further distinguish and confirm the varying chromatographic peaks in sun-dried or sulfur-fumigated LJF samples, LC-ESI/MS^n^ analysis was performed on an Agilent 1200 HPLC system (Agilent, Waldbronn, Germany) coupled to a diode array detector and mass spectrometer (Agilent 6320 Ion Trap). The HPLC conditions were the same as those described above. The LC effluent was introduced into an electrospray ionization source after a post-column split ratio of 1:4. The parameters for LC-MS analysis were as follows: dry temperature, 350 °C; nebulizer, 50.0 psi; and dry gas, 12.0 mL/min. Full-scan mass spectra were acquired in the positive and negative ionization modes over the range *m/z* 100–1000.

### 3.4. Quantitative HPLC Analysis

The quantitative analysis of nine compounds in sun-dried and sulfur-fumigated LJF samples, including chlorogenic acid (**3**), secologanic acid (**6**), loganin (**8**), sweroside (**9**), rutin (**15**), luteoloside (**17**), isochlorogenic acid A (**20**), isochlorogenic acid B (**19**) and isochlorogenic acid C (**21**), was carried out by HPLC-UV. The same HPLC system, chromatographic column and mobile phase were used as described in Section 3.3. The linear gradient was optimized as follows: 0–40 min (10%–14% B), 40–65 min (14%–22.5% B), 65–75 min (22.5%–40% B). The flow rate was at 0.8 mL/min, and the detection wavelength was set at 254 nm for secologanic acid (**6**), loganin (**8**) and sweroside (**9**) and 350 nm for chlorogenic acid (**3**), rutin (**15**), luteoloside (**17**), isochlorogenic acid A (**20**), isochlorogenic acid B (**19**) and isochlorogenic acid C (**21**), respectively.

### 3.5. Determination of Residual Sulfur Dioxide

The residual sulfur dioxide in the sun-dried and sulfur-fumigated LJF samples was determined by iodine titration according to the current Chinese Pharmacopoeia [13].

### 3.6. The Antioxidant Capacity

The DPPH assay was performed according to the method described by Wang *et al.* [11]. In brief, 2 mL of LJF extract solution and 2 mL of DPPH (200 µmol/L) were mixed. After 30 min, the absorbance was measured at 517 nm at room temperature by using an ultraviolet spectrophotometer (T6-new century, Beijing Purkinje General Instrument Co., Ltd., Beijing, China). All samples were tested in triplicate. The scavenging ability against DPPH was expressed as a percent of the suppression of the DPPH radical, and the effective concentration (EC_50_) was determined for antioxidants. All results were expressed as the mean ± standard deviation (SD), and the data were calculated by SPSS version 17.0 with a significance level of 0.05.

### 3.7. Statistical Analysis

In order to demonstrate the resemblance and difference of the sulfur-fumigated and sun-dried samples, as well as the easily-influenced constituents during the sulfur fumigation process, PCA and PLS were applied by the software, SIMCA-P+11 (Umetrics AB, Umea, Sweden). The bivariate correlation analysis was carried out by the software, SPSS 17.0, to find out the possible relationship of the EC_50_ values and the contents of the main compounds in LJF samples.

## 4. Conclusions

In this study, the influence of sulfur fumigation on the chemical constituents and antioxidant activity of buds of *Lonicera japonica* was comprehensively investigated. This was the first time that secologanic acid along with the other eight commonly-analyzed constituents in LJF samples by HPLC was determined simultaneously, and the method was also validated. Secologanic acid was destroyed, and its sulfonates were generated through esterification or ring-opening, followed by an addition reaction, whereas the caffeoylquinic acids were protected from being oxidized due to the inactivity of PPO during the sulfur fumigation processing. However, this process had no obvious effect on the contents of the flavonoid compounds. In addition, the residual sulfur dioxide in sulfur-fumigated samples was significantly higher than that of sun-dried samples, which might increase the potential incidence of toxicity for humans. Meanwhile, compared with sun-dried LJF samples, sulfur-fumigated LJF samples have significantly stronger antioxidant activity, which could be attributed to the joint effect of the protected phenolic acids and flavonoids, as well as newly-generated iridoid sulfonates, with more antioxidant activity during the sulfur fumigation process.

## Supplementary Materials

PCA and PLS plots of qualitative HPLC data for sun-dried and sulfur-fumigated can be accessed at: http://www.mdpi.com/1420-3049/19/10/16640/s1. The linearity equation, LOD and LOQ for nine marker compounds, as well as the results of method validation are shown in Table S1 and Table S2.

## Supplementary Files

Supplementary File 1
