# Supplementary material for: Influence of Sulfur Fumigation on the Chemical Constituents and Antioxidant Activity of Buds of Lonicera japonica
Source: Molecules. 2014 Oct 15;19(10):16640–55. doi: 10.3390/molecules191016640 (PMC6271180; doi:10.3390/molecules191016640)
Supplement: Supplementary File 1 [file molecules-19-16640-s001.pdf]

Supplementary Materials

Figure S1. PCA (principal component analysis) and PLS (partial least squares) plots of the qualitative HPLC data of sulfur-fumigated (Nos. 3–8) and sun-dried (Nos. 11–16) LJF. (**a**) PCA; (**b**) PLS, Class 1 (sulfur-fumigated), Class 2 (sun-dried); (**c**) PLS-DA (discriminant analysis)-VIP (variable importance in the projection).

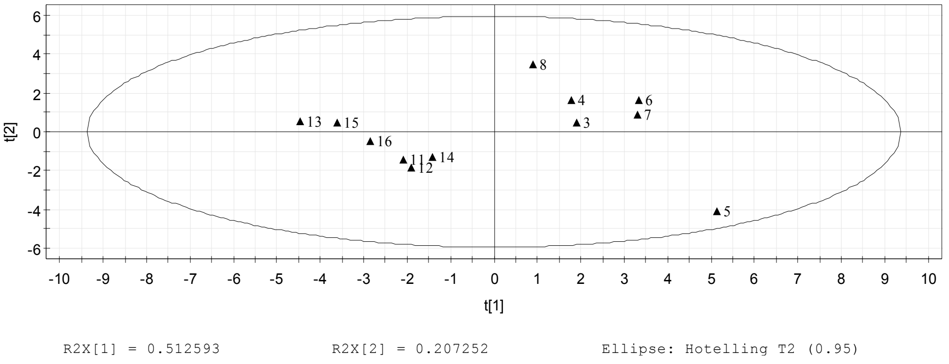

(**a**)

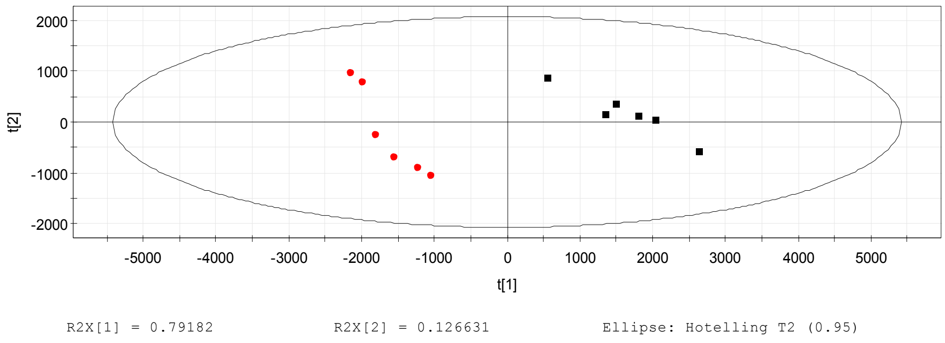

(**b**)

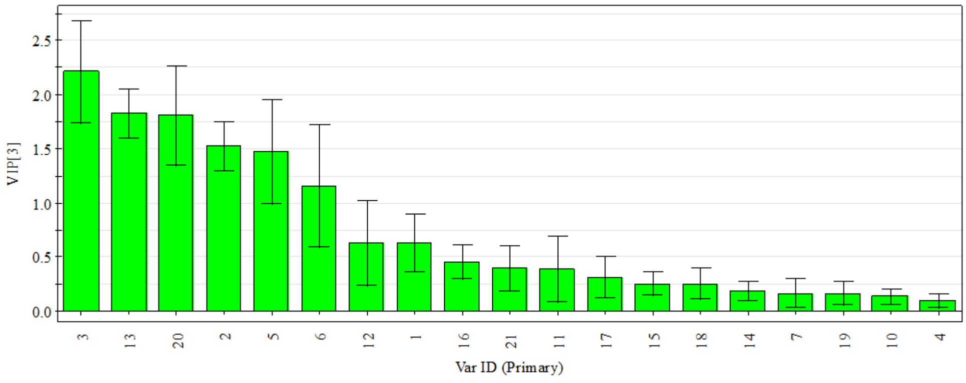

(**c**)

The linearity of the concentration (X, mg/L) *versus* peak area (Y) was investigated for nine marker compounds. The results expressed as the values of the correlation coefficient (R^2) are shown in Table S1. Their LODs and LOQs were separately determined as 0.0525–0.236 mg/L and 0.175–0.625 mg/L at an S/N of 3 and 10, respectively. For the validation of the assay procedure, the results of precision and repeatability were indicated by RSDs that were less than 3.92%, for all determined compounds (n = 6). The stability test suggested that secologanic acid was stable within 24 h, and the other eight markers were stable within 48 h. The average recoveries were in the range from 95.35% to 104.7% with an RSD less than 4.76%, indicating that the developed method was accurate for the determination of nine compounds in LJF samples (Table S2).

Table S1. Precision, repeatability, stability, regression equations, LODs and LOQs for nine compounds.

No.	$Y = aX + b$	R^2	Range (mg/L)	Precision RSD (%)	Repeatability		Stability [a] RSD (%)	Stability [b] RSD (%)	LOD (mg/L, 10 µL)	LOQ (mg/L, 10 µL)
					Contents (%)	RSD (%)				
**3**	$Y = 17376X - 37427$	0.9999	5.25–336	0.55	0.840 ± 0.0089	1.06	0.291	0.628	0.236	0.525
**6**	$Y = 9100.9X + 7087.1$	0.9999	7.50–480	1.3	1.89 ± 0.0210	1.11	3.12	5.44	0.180	0.600
**8**	$Y = 10296X - 2905$	0.9999	0.625–40	2.9	0.0192 ± 0.0008	3.92	1.75	3.02	0.156	0.625
**9**	$Y = 18768X - 2482.7$	0.9999	0.78–50	2.9	0.0687 ± 0.0011	1.54	1.77	2.95	0.117	0.391
**15**	$Y = 26068X - 3330.4$	0.9999	0.75–48	0.43	0.0522 ± 0.0012	2.22	0.213	0.436	0.112	0.375
**17**	$Y = 49459X - 10711$	0.9999	0.87–56	0.51	0.0578 ± 0.0004	0.649	0.193	0.568	0.0525	0.175
**19**	$Y = 22848X - 5155.5$	0.9999	0.625–40	0.65	0.0276 ± 0.0006	2.33	0.432	0.622	0.0937	0.312
**20**	$Y = 22823X - 31104$	0.9999	1.25–200	0.45	1.39 ± 0.0099	0.710	0.179	0.615	0.141	0.469
**21**	$Y = 26278X - 12024$	0.9999	1.09–70	0.43	0.148 ± 0.0013	0.848	0.218	0.517	0.109	0.273

[a] The peak areas of nine compounds were recorded in 24 h after the preparation of the sample solution; [b] the peak areas of nine compounds were recorded in 48 h after the preparation of the sample solution.

Table S2. Accuracy of the HPLC method for the determination of the investigated compounds (n = 3).

Compounds	Original (µg)	Added (µg)	Found (µg)	Recovery (%)	RSD (%)
**3**	840	1176	1129 ± 15.89	95.96	1.41
		840	806 ± 7.20	95.99	0.89
		504	481 ± 5.01	95.35	1.04
**6**	1894	2652	2697 ± 24.49	101.7	0.88
		1894	1867 ± 46.98	98.61	2.54
		1136	1084 ± 1.36	95.35	0.13
**9**	69	96	94 ± 2.27	98.14	2.40
		69	69 ± 1.43	100.4	2.06
		41	41 ± 1.03	99.91	2.50
**15**	52	73	71 ± 0.018	96.66	1.83
		52	51 ± 0.018	98.08	1.77
		31	31 ± 0.027	98.53	2.74
**17**	58	81	81 ± 0.84	99.83	1.04
		58	58 ± 1.17	99.60	2.03
		35	36 ± 0.41	104.6	1.13
**19**	28	39	39 ± 1.84	99.35	4.76
		28	28 ± 0.56	103.0	1.98
		17	17 ± 0.91	101.8	1.96
**20**	1390	1945	2037 ± 15.1	104.7	0.75
		1390	1409 ± 26.9	101.4	1.90
		834	833 ± 6.94	99.96	0.83
**21**	148	207	205 ± 2.51	98.46	1.23
		148	142 ± 0.57	95.43	0.40
		89	89 ± 3.50	100.4	4.75
